# Genetic Plurality of OXA/NDM-Encoding Features Characterized From *Enterobacterales* Recovered From Czech Hospitals

**DOI:** 10.3389/fmicb.2021.641415

**Published:** 2021-02-09

**Authors:** Katerina Chudejova, Lucie Kraftova, Vittoria Mattioni Marchetti, Jaroslav Hrabak, Costas C. Papagiannitsis, Ibrahim Bitar

**Affiliations:** ^1^Department of Microbiology, Faculty of Medicine, University Hospital in Pilsen, Charles University, Pilsen, Czechia; ^2^Biomedical Center, Faculty of Medicine, Charles University, Pilsen, Czechia; ^3^Department of Microbiology, University Hospital of Larissa, Larissa, Greece

**Keywords:** OXA-244, OXA-181, NDM-1, NDM-5, mobile genetic elements

## Abstract

The aim of this study was to characterize four *Enterobacterales* co-producing NDM- and OXA-48-like carbapenemases from Czech patients with travel history or/and previous hospitalization abroad. *Klebsiella pneumoniae* isolates belonged to “high risk” clones ST147, ST11, and ST15, while the *Escherichia coli* isolate was assigned to ST167. All isolates expressed resistance against most β-lactams, including carbapenems, while retaining susceptibility to colistin. Furthermore, analysis of WGS data showed that all four isolates co-produced OXA-48- and NDM-type carbapenemases, in different combinations (Kpn47733: *bla*_NDM–__5_ + *bla*_OXA–__181_; Kpn50595: *bla*_NDM–__1_ + *bla*_OXA–__181_; Kpn51015: *bla*_NDM–__1_ + *bla*_OXA–__244_; Eco52418: *bla*_NDM–__5_ + *bla*_OXA–__244_). In Kpn51015, the *bla*_OXA–__244_ was found on plasmid p51015_OXA-244, while the respective gene was localized in the chromosomal contig of *E. coli* Eco52418. On the other hand, *bla*_OXA–__181_ was identified on a ColKP3 plasmid in isolate Kpn47733, while a *bla*_OXA–__181_-carrying plasmid being an IncX3-ColKP3 fusion was identified in Kpn50595. The *bla*_NDM–__1_ gene was found on two different plasmids, p51015_NDM-1 belonging to a novel IncH plasmid group and p51015_NDM-1 being an IncF_*K*__1_-FIB replicon. Furthermore, the *bla*_NDM–__5_ was found in two IncFII plasmids exhibiting limited nucleotide similarity to each other. In both plasmids, the genetic environment of *bla*_NDM–__5_ was identical. Finally, in all four carbapenemase-producing isolates, a diverse number of additional replicons, some of these associated with important resistance determinants, like *bla*_CTX–M–__15_, *arr-2* and *ermB*, were identified. In conclusion, this study reports the first description of OXA-244-producing *Enterobacterales* isolated from Czech hospitals. Additionally, our findings indicated the genetic plurality involved in the acquisition and dissemination of determinants encoding OXA/NDM carbapenemases.

## Introduction

The increased incidence of multidrug-resistant (MDR) Gram-negative bacteria worldwide over the last decade has been worrisome (Bassetti et al., 2019). Carbapenems are considered the drug of choice in treating such infections. However, the increased usage of these antibiotics has led to the emergence of carbapenem-resistant strains (Roberts et al., 2020). Center for Disease Control (CDC) considers carbapenem-resistant *Enterobacterales* (CRE) as a serious global threat to patient health that limits treatment options, especially in chronically ill patients in intensive care units (ICU) and long-term care facilities (McConville et al., 2017; Gupta et al., 2019). Resistance to carbapenems is caused by various mechanisms, such as porin loss, increased efflux pump activity and most importantly the production of carbapenemases (Ye et al., 2018).

Acquired carbapenem-hydrolyzing β-lactamases are enzymes of the Ambler class A KPC type, class B type, including IMP-, VIM-, and NDM-like metallo-β-lactamases (MβLs), or the class D OXA-48 type. However, in *Enterobacterales*, the most clinically significant metallo-β-lactamases are the NDM-like enzymes (Nordmann et al., 2011). NDM-like enzymes efficiently hydrolyze broad range of β-lactam antibiotics, including penicillins, cephalosporins, and carbapenems, with the exception of monobactams such as aztreonam (Nordmann and Poirel, 2014). In 2008, NDM-1 was reported for the first time (Yong et al., 2009) and, shortly after, 24 distinct NDM enzymes have been described worldwide, most of them originating from India, China, Nepal, or Near East (Hornsey et al., 2011; Kaase et al., 2011; Nordmann et al., 2012; Williamson et al., 2012; Rogers et al., 2013; Tada et al., 2013; Wang et al., 2014; Wu et al., 2019). Compared to NDM-1, NDM-5 MβL has two amino acid substitutions (Val88Leu and Met154Leu) (Hornsey et al., 2011). NDM-5 was reported, for the first time, from a clinical *Escherichia coli* strain in the United Kingdom (Hornsey et al., 2011). In the Czech Republic, NDM-1 and NDM-5 enzymes were reported for the first time in 2011 and in 2016, respectively (Hrabak et al., 2012; Paskova et al., 2018).

Also, OXA-48-producing *Enterobacterales* pose an important public threat, mainly due to their challenging detection and the rapid horizontal transfer of pOXA-48-like plasmids (Skalova et al., 2017). OXA-48-like enzymes hydrolyze penicillins at a high level and carbapenems at a low level, sparing broad-spectrum cephalosporins, and are not susceptible to β-lactamase inhibitors (Poirel et al., 2004). Since its first report in Turkey in 2004, 11 variants with few amino acid substitutions or deletions emerged globally (Bakthavatchalam et al., 2016; Mairi et al., 2018). In 2006–2007, OXA-181 was reported for the first time in India and since then it is considered one of the most disseminated OXA-48-like enzymes worldwide especially in patients with travel history to the Indian continent (Castanheira et al., 2011; Rojas et al., 2017). In 2011, OXA-244 was reported for the first time in Spain, since then there has been only limited number of reports, indicating limited dissemination (Oteo et al., 2013; Fursova et al., 2015; Potron et al., 2016; van Hattem et al., 2016). Recently, co-production of NDM- and OXA-48-like carbapenemase has been increasingly described, especially in patients with travel history to Italy (Marchetti et al., 2019), South Korea (Baek et al., 2019), Turkey (Otlu et al., 2018), Singapore (Balm et al., 2013) and the United States (Doi et al., 2014; Contreras et al., 2020).

Thus, the aim of this study was to genomically characterize four isolates (three *Klebsiella pneumoniae* and one *E. coli*) co-producing NDM- and OXA-48-like carbapenemases from Czech patients with travel history or/and previous hospitalization abroad.

## Materials and Methods

### Case Presentations

The first case was reported, in December 2018, from a Czech patient admitted to the Neuro Intensive Unit Care (ICU) of the Military University Hospital in Prague for head injury and concussion. The patient had traveled shortly before admission to India, where he/she was hospitalized due to a motorcycle accident, and then transferred back to Prague. A rectal swab was collected, highlighting a *K. pneumoniae* isolate (Kpn47733) co-producing NDM- and OXA-48-like carbapenemases.

The second case referred to an inpatient of the Rehabilitation Unit of the Malvazinky Clinic in Prague, who underwent an orthopedic surgery for hip replacement in May 2019. During rehabilitation, the patient developed a urinary tract infection. Urine culture confirmed the presence of a *K. pneumoniae* isolate (Kpn50595), coproducing NDM- and OXA-48-like carbapenemases. The patient had a travel history 2 weeks before admission (April 2019) to Mauritius, but didn’t have any history of hospitalization there.

The third case was a patient admitted to Hepatogastroenterology Unit of the Institute of Clinical and Experimental Medicine in Prague for bile duct obstruction, in June 2019. As a part of the screening process, a rectal swab was performed, and culture highlighted the presence of a *K. pneumoniae* isolate (Kpn51015), co-producing NDM- and OXA-48-like enzymes. The patient had a travel history to Egypt in December 2018, without hospitalizations.

The fourth case was reported in August 2019, when a female patient was admitted to the Nephrology Ambulatory of the University Hospital of Ostrava, showing urinary tract infection symptoms. Urine sample culture identified the presence of an *E. coli* isolate (Eco52148), co-producing NDM- and OXA-48-like enzymes. The patient’s hospitalization history showed that she had kidney transplantation shortly before being admitted. Additionally, the patient was recently repatriated from northern part of Africa.

### Carbapenem Production and Susceptibility Testing

These four isolates, mentioned above, were selected to be further characterized, since they were the only *Enterobacterales*, co-producing NDM- and OXA-48-like enzymes, referred from local microbiological laboratories to our lab, during 2018–2019. Species identification of the four strains was performed using matrix-assisted laser desorption ionization-time of flight mass spectrometry (MALDI-TOF MS) through MALDI Biotyper software (Bruker Daltonics, Bremen, Germany). MALDI-TOF MS meropenem hydrolysis assay was used to confirm carbapenem production (Rotova et al., 2017). Production of carbapenemases (metallo-β-lactamase, OXA-48 and KPC) was assessed using the double-disc synergy test with EDTA, temocillin disc test and phenylboronic acid test (Lee et al., 2003; Doi et al., 2008; Glupczynski et al., 2012). The isolates were screened by PCR for the presence of *bla*_NDM_-like *bla*_VIM_-like, *bla*_IMP_-like, *bla*_KPC_-like and *bla*_OXA–__48_-like genes (Papagiannitsis et al., 2015). Antimicrobial susceptibility was performed using broth microdilution according to European Committee on Antimicrobial Susceptibility Testing (EUCAST) guidelines. Susceptibility to fosfomycin was performed using agar dilution based on EUCAST guidelines. Susceptibility data were interpreted according to the criteria (version 10.0) of the EUCAST^1^.

### Transfer of Carbapenemase-Encoding Genes

The conjugal transfer of carbapenemase-encoding genes was tested in liquid medium using the *E. coli* A15 strain (Azd^*R*^) as recipient. Transconjugants were selected on MacConkey agar (Scharlab, SL, Barcelona, Spain) plates containing sodium azide (100 mg/L) (Sigma-Aldrich, St. Louis, MO, United States) and ampicillin (100 mg/L) (Sigma-Aldrich). The presence of *bla*_NDM_-like and *bla*_OXA–__48_-like was confirmed by PCR.

### Whole-Genome Sequencing and Analysis

Genomic DNA was extracted from the four clinical isolates using NucleoSpin Microbial DNA kit (Macherey-Nagel, Germany). Whole genome sequencing (WGS) was performed on the Sequel I platform (Pacific biosciences, Menlo Park, CA, United States). Microbial multiplexing protocol was used for the library preparation according to the manufacturer instructions for Sheared DNA. DNA shearing was performed using the Megaruptor 2 (Diagenode, Liege, Belgium) using long hydropores producing 15kb long inserts. No size selection was performed during the library preparation. Microbial Assembly pipeline offered by the SMRT Link v8.0 software was used to perform the assembly and circularization with minimum seed coverage of 30×. Assembled sequences were annotated using the NCBI Prokaryotic Genome Annotation Pipeline (PGAP). Antibiotic resistant genes, plasmid replicons, mobile elements and multilocus sequence types (MLST) were determined through uploading the assembled sequences to ResFinder 4.1 and CARD (Zankari et al., 2012; Alcock et al., 2020), PlasmidFinder (Carattoli et al., 2014), ISfinder (Siguier et al., 2006), and MLST 2.0 (Larsen et al., 2012), respectively. Comparative genome alignment was done using Mauve v.2.3.1.^2^ and BLAST Ring Image Generator (BRIG) (Alikhan et al., 2011). Diagrams and gene organization were sketched using Inkscape 0.92.4^3^.

### Nucleotide Sequence Accession Numbers

The nucleotide sequences of the genomes and plasmids of Kpn47733, Kpn50595, Kpn51015, and Eco52148 has been uploaded to GenBank under the accession numbers CP050360-CP050370, CP050371-CP050375, CP050376-CP050381, and CP050382-CP050384 respectively.

## Results

All isolates expressed resistance to ampicillin, ciprofloxacin, piperacillin, piperacillin-tazobactam, cefotaxime, meropenem, and ertapenem, while retaining susceptibility to colistin. Moreover, all isolates, except for Eco52148, showed resistance against gentamicin, amikacin, netilmicin and tobramycin. On the other hand, isolates Eco52148 and Kpn47733 showed resistance to tetracycline (Table 1).

**TABLE 1:** Susceptibility profiles of *Enterobacterales*, co-producing NDM- and OXA-48-like carbapenemases, isolates collected in Czech hospitals, during the study.

Isolate	ST	MIC (mg/L)														
		Amp	Pip	Tzp	Ctx	Caz	Mem	Etp	Gm	Amk	Tm	Net	Tet	Tgc	Col	Fos
*K. pneumoniae* Kpn47733	147	>128	>128	>128	>8	>16	>16	>2	>32	>64	>8	>16	16	1	0.25	128
*K. pneumoniae* Kpn50595	11	>128	>128	>128	>8	>16	16	>2	>32	>64	>8	>16	2	0.5	0.25	32
*K. pneumoniae* Kpn51015	15	>128	>128	>128	>8	>16	8	>2	>32	>64	>8	>16	1	0.25	0.25	64
*E. coli* Eco52148	167	>128	>128	>128	>8	>16	4	>2	4	64	0.5	1	>32	0.25	0.25	0.5

*MIC, minimum inhibitory concentration; Amp, ampicillin; Pip, piperacillin; Tzp, piperacillin-tazobactam; Ctx, cefotaxime; Caz, ceftazidime; Mem, meropenem; Etp, ertapenem; Gm, gentamicin; Amk, amikacin; Tm, tobramycin; Net, netilmicin; Tet, tetracycline; Tgc, tigecycline; Col, colistin; Fos, fosfomycin.*

WGS performed on the Sequel I platform and assembly performed on Microbial Assembly pipeline resulted in complete, closed chromosomes and plasmids shown in Table 2. WGS revealed that *K. pneumoniae* isolates Kn47733, Kpn50595, and Kpn51015 belonged to sequence types (STs) 147, 11 and 15, respectively. All these three STs have been considered as “high risk” clones (Woodford et al., 2011). The *E. coli* isolate Eco52418 was assigned to ST167. Several studies have reported the association of ST167 *E. coli* with the dissemination of resistance genes, especially of the carbapenemase-encoding gene *bla*_NDM–__5_ (Mani et al., 2017; Sánchez-Benito et al., 2017; Sun et al., 2018; Xu et al., 2019).

**TABLE 2:** WGS data of *Enterobacterales*, co-producing NDM- and OXA-48-like carbapenemases, isolates recovered from Czech hospitals.

Isolate	ST	Replicons	Size	Plasmid name	Inc group	Carbapenemase encoding-genes	Other resistance genes	GenBank accession no.
Kpn47733	147	Chromosome	5401559 bp	–	–	–	*bla*_CTX–M–15_, *bla*_SHV–11_, *oqxA*, *oqxB*, *fosA*	CP050360
		Plasmid	103085 bp	p47733_NDM-5	IncFII	*bla*_NDM–__5_	*aadA2*, *rmtB*, *bla*_TEM–__1__B_, *erm*(B), *mph(*A), *sul1*, *dfrA12*	CP050367
		Plasmid	6812 bp	p47733_OXA-181	ColKP3	*bla*_OXA–__181_	–	CP050368
		Plasmid	119981 bp	p47733_CTX-M-15	R	–	*rmtF*, *bla*_CTX–M–__15_, *mph*(A), *catA2*, *aac*(6’*)-lb-cr*, *qnrB1*, *drfA12*, *dfrA14*	CP050364
		Plasmid	107451 bp	p47733_ARR-2	IncFII	–	*rmtF*, *aac*(6’)*-lb-cr*, *arr-2*	CP050361
		Plasmid	115360 bp	p47733_IncFIB	IncFIB	–	–	CP050365
		Plasmid	2101 bp	p47733_Col_BS512	Col	–	–	CP050362
		Plasmid	1546 bp	p47733_Col_MG828	Col	–	–	CP050363
		Plasmid	2056 bp	p47733_Col_PVC	Col	–	–	CP050370
		Plasmid	4715 bp	p47733_S	NT	–	–	CP050369
		Plasmid	55119 bp	p47733_L	NT	–	–	CP050366
Kpn50595	11	Chromosome	5339674 bp	–	–	–	*bla*_SHV–11_, *fosA*, *oqxA*, *oqxB*	CP050371
		Plasmid	193462 bp	p50595_NDM-1	IncFIB-IncFII	*bla*_NDM–__1_	*aac*(3)*-lla*, *aac*(6’)*-lb-cr*, *rmtF*, *bla*_CTX–M–__15_, *catB*, *arr-2*	CP050374
		Plasmid	51140 bp	p50595_OXA-181	IncX3-ColKP3	*bla*_OXA–__181_	*qnrS1*	CP050375
		Plasmid	76387 bp	p50595_ERM	IncFII	–	*erm*(B), *mph*(A)	CP050372
		Plasmid	127925 bp	p50595	IncFII-IncFIB	–	–	CP050373
Kpn51015	15	Chromosome	5306856 bp	–	–	–	*bla*_SHV–28_, *fosA*, *oqxA*, *oqxB*	CP050376
		Plasmid	353810 bp	p51015_NDM-1	IncFIB-IncHI1B	*bla*_NDM–__1_	*aph*(3’)*-la*, *aph*(3’)*-VI*, *armA*, *mph*(A), *mph*(E), *msr*(E), *qnrS1*, *sul1*, *sul2*, *dfrA5*	CP050380
		Plasmid	71402 bp	p51015_OXA-244	IncFII	*bla*_OXA–__244_	–	CP050381
		Plasmid	225540 bp	p51015_CTX-M-15	IncFII-IncFIB	–	*aac*(3)*-lld*, *aac*(6’)*-lb-cr*, *aph*(3”)*-lb*, *aph*(3’)*-la*, *aph*(6)*-ld*, *bla*_CTX–M–__15_, *bla*_OXA–__1_, *bla*_TEM–__1__B_, *mph*(A), *catB3*, *sul2*	CP050379
		Plasmid	18651 bp	p51015_ColRNAI	ColRNAI	–	–	CP050378
		Plasmid	1565 bp	p51015	NT	–	–	CP050377
Eco52148	167	Chromosome	4859628 bp	–	–	*bla*_*OXA–244*_	*mdf*(A)	CP050382
		Plasmid	121872 bp	p52148_NDM-5	IncFII-IncFIA	*bla*_NDM–__5_	*aadA2*, *mph*(A), *sul1*, *tet*(A), *dfrA12*	CP050384
		Plasmid	4081 bp	p52148	NT	–	–	CP050383

*NT, non-typed.*

Furthermore, analysis of WGS data showed that all four isolates carried different combinations of carbapenemase-encoding genes (Kpn47733: *bla*_NDM–__5_ + *bla*_OXA–__181_; Kpn50595: *bla*_NDM–__1_ + *bla*_OXA–__181_; Kpn51015: *bla*_NDM–__1_ + *bla*_OXA–__244_; Eco52418: *bla*_NDM–__5_ + *bla*_OXA–__244_) (Table 2). Noteworthy, based on our knowledge, this is the first report of *Enterobacterales*, carrying *bla*_OXA–__244_ gene, isolated from the Czech Republic. Additionally, all isolates exhibited a wide variety of resistance genes conferring resistance to β-lactams, aminoglycosides, sulfonamides, macrolides, lincosamides, streptogramin b, fosfomycin (low-level resistance), fluoroquinolones, chloramphenicol, tetracyclines, and/or rifampicin (Table 2).

The carbapenem resistance phenotypes of all clinical strains were transferred to azide-resistant *E. coli* A15 by conjugation (Supplementary Table 1). For isolates Kpn47733, Kpn50595 and Kpn51015, all transconjugants carried both carbapenemase-encoding genes, while only the *bla*_NDM–__5_ gene was identified in the transconjugants of the *E. coli* isolate Eco52148.

Analysis of contigs carrying carbapenemase-encoding genes showed that, in isolate Kpn51015, the *bla*_OXA–__244_ gene was found on a plasmid (p51015_OXA-244) of 71402 bp in size, while the respective gene was localized in the chromosomal contig of *E. coli* isolate Eco52418. In both isolates, the *bla*_OXA–__244_ gene was bounded by two copies of IS*1* insertion sequence in parallel orientation (Supplementary Figure 1), forming a composite transposon, named Tn*51098*. In the *E. coli* isolate Eco52418, the Tn*51098* transposon was integrated into an open reading frame (ORF) encoding an HNH endonuclease (nts 576701 to 580010 in GenBank accession no. CP050382), as described previously (Potron et al., 2016; Hoyos-Mallecot et al., 2017). Direct repeats of 9 bp (TGAATTGCT) were found at the boundaries of the *bla*_OXA–__244_-carrying composite transposon, suggesting its transposition into the *E. coli* chromosome. However, unlike the isolate Eco52418, an ORF encoding a LysR transcriptional regulator wasn’t found between *bla*_OXA–__244_ (downstream) and IS*1*, in plasmid p51015_OXA-244. Plasmid p51015_OXA-244, which belonged to the incompatibility group FII (IncFII), exhibited extensive similarity with IncFII plasmids from *E. coli* strains D181 and F5176C6 (GenBank accession nos. CP024250 and CP024669, respectively) (Supplementary Figure 2A). Unlike p51015_OXA-244, those plasmids were negative for the presence of *bla*_OXA–__244_ gene. Among p51015_OXA-244, no resistance genes other than *bla*_OXA–__244_ were identified.

On the other hand, the *bla*_OXA–__181_ carbapenemase-encoding gene was identified on a ColKP3 plasmid (p47733_OXA-181) of 6812 bp in size, in isolate Kpn47733, while a *bla*_OXA–__181_-carrying plasmid (p50595_OXA-181; 51140 bp), being an IncX3-ColKP3 fusion, was identified in isolate Kpn50595. In both plasmids, the *bla*_OXA–__181_ genes were surrounded by identical sequences (Supplementary Figure 3). In comparison with the archetypal ColE2-type plasmid pKP3-A carrying *bla*_OXA–__181_ (Potron et al., 2011), p47733_OXA-181 was composed only of *repA* and *bla*_OXA–__181_ genes (Supplementary Figure 2A). The *mob* genes, encoding proteins that form a plasmid mobilization system, were not found in plasmid p47733_OXA-181. One additional difference between the two plasmids was the presence of Tn*5403* transposon in p47733_OXA-181. The Tn*5403* transposon has been previously found in *bla*_NDM–__1_-positive IncN2 plasmids, like plasmid pJN24NDM1 characterized from a ST405 *E. coli* from China (Hao et al., 2019). On the other hand, plasmid p50595_OXA-181 was almost identical to plasmids p1-Ec-BERN-042 (100% coverage, 99.99% identity; GenBank accession no. CP042935) and pOXA181_29144 (100% coverage, 99.99% identity) (Supplementary Figure 2A). Plasmid pOXA181_29144 was previously characterized from a ST18 *K. pneumoniae* strain (Kpn-29144) isolated, in 2015, in the Czech Republic (Skalova et al., 2017). Similar to pOXA181_29144, which was transferable by conjugation (Skalova et al., 2017), a complete *tra* locus was found in the sequence of p50595_OXA-181. Also, the *qnrS1* gene, conferring low-level resistance to fluoroquinolones, was identified in the sequence of p50595_OXA-181.

**FIGURE 1:**
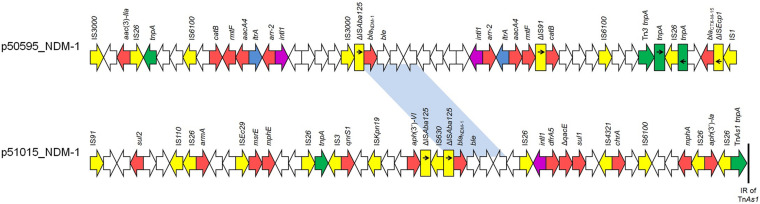
Linear maps of the multidrug resistance regions (MDRs), carrying *bla*_NDM–__1_ genes. Arrows show the direction of transcription of open reading frames (ORFs), while truncated ORFs appear as rectangles (arrows within rectangles indicate the direction of transcription). Resistance genes are shown in red. IS elements and transposases are shown in yellow and green, respectively. *intI1* genes are shaded purple. The remaining genes are shown in white. Homologous segments (representing ≥99% sequence identity) are indicated by light blue shading.

The *bla*_NDM–__1_ carbapenemase-encoding gene was found on two different plasmid types (Supplementary Figure 4). In isolate Kpn51015, a *bla*_NDM–__1_-positive plasmid (p51015_NDM-1) of 353810 bp in size was identified, while a 193462-bp plasmid (p50595_NDM-1) carrying *bla*_NDM–__1_ was found in isolate Kpn50595. Plasmid p51015_NDM-1 exhibited extensive similarity to *bla*_NDM–__5_-carrying plasmid pKpvST383L (99% coverage, 99% identity; GenBank accession no. CP034201) (Supplementary Figure 2B), characterized from a ST383 *K. pneumoniae* recovered in London. p51015_NDM-1 belonged to a novel IncH plasmid group, harboring FIB and HIB replicons previously observed in *bla*_NDM–__1_-carrying plasmid pNDM-MAR characterized from a ST15 *K. pneumoniae* isolated in Morocco (Villa et al., 2012). In the MDR of p51015_NDM-1, beside *bla*_NDM–__1_, *aph*(3’)*-la*, *aph*(3’)*-VI*, *armA*, *mph*(A), *mph*(E), *msr*(E), *qnrS1*, *sul1*, *sul2*, and *dfrA5* genes, conferring resistance to aminoglycosides, macrolides, streptogramin b, fluoroquinolones, sulfonamides and trimethoprim, were found (Figure 1). Additionally, plasmid p51015_NDM-1 carried tellurium resistance genes (*terZABCDEF*), commonly associated with this plasmid family (Zingali et al., 2020). Unlike p51015_NDM-1, p50595_NDM-1 was an IncF_*K*__1_-FIB plasmid being a fusion derivative of previously characterized plasmids, like pUCLAOXA232-3, p51015_CTX_M_15, pKPX-1, and pGR-1870 (GenBank accession no. CP012564, CP050379, AP012055 and KF874498) (Supplementary Figure 2B). In p50595_NDM-1, the *bla*_NDM–__1_ gene was found in a genetically distant MDR region than observed in plasmid p51015_NDM-1. The MDR region of p50595_NDM-1 also contained *bla*_CTX–M–__15_, *aac*(3)*-IIa*, *aacA4* (2 copies), *rmtF* (2 copies), *catB* (2 copies) and *arr-2* (2 copies) genes conferring resistance to β-lactams, aminoglycosides, chloramphenicol and rifampicin (Figure 1). Additionally, plasmid backbone of p50595_NDM-1 included an arsenate resistance region. In both *bla*_NDM–__1_-carrying plasmids, several insertion sequences (ISs) that could be involved in the organization of their MDR regions were found.

**FIGURE 2:**
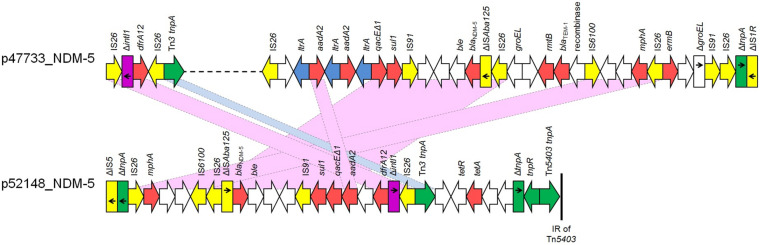
Linear maps of the multidrug resistance regions (MDRs), carrying *bla*_NDM–__5_ genes. Arrows show the direction of transcription of open reading frames (ORFs), while truncated ORFs appear as rectangles (arrows within rectangles indicate the direction of transcription). Resistance genes are shown in red. IS elements and transposases are shown in yellow and green, respectively. *intI1* genes are shaded purple. The remaining genes are shown in white. Homologous segments (representing ≥99% sequence identity) are indicated by light blue shading, while pink shading shows inverted homologous segments.

Furthermore, the *bla*_NDM–__5_ gene was found in two IncFII plasmids, p47733_NDM-5 and p52418_NDM-5, which contained complete *tra* operons. The FII (allele 2) plasmid p47733_NDM-5 (103085 bp), found in *K. pneumoniae* Kpn47733, showed extensive similarity to the *bla*_NDM–__5_-carrying plasmid pCRKP-2297_2 (99% coverage, 99.96% identity; GenBank CP024836) (Supplementary Figure 2B) characterized from *K. pneumoniae* strain CRKP-2297 recovered in South Korea. In p47733_NDM-5, the *bla*_NDM–__5_ gene was found in a MDR region of 31159 bp (nts 38315-69473 in GenBank accession no. CP050367) in size. This MDR region, which was bounded by two copies of IS*26* element, also included *bla*_TEM–__1_, *aadA2* (2 copies), *sul1*, *rmtB*, *mphA* and *ermB* genes conferring resistance to β-lactams, aminoglycosides, sulfonamides, macrolides, lincosamides, and streptogramin b. An additional resistance gene, *dfrA12*, being the unique gene cassette of a class 1 integron was identified ∼26 Kb upstream of the p47733_NDM-5 MDR region (Figure 2). While both *bla*_NDM–__5_-carrying plasmids belonged to IncFII group, the FII (allele 36) plasmid p52148_NDM-5 (121872 bp), found in *E. coli* isolate Eco52148, harbored a second replicon, FIA (allele 4) (nts 85531-86286 in GenBank accession no. CP050384). Plasmid p52148_NDM-5 exhibited limited nucleotide similarity (53% coverage, 100% identity) against p47733_NDM-5. However, it was highly similar to *bla*_NDM–__5_-carrying plasmid p1ESCUMpO83_CORR (100% coverage, 99.98 identity; GenBank accession no. CP033159) (Supplementary Figure 2B) characterized from a pathogenic *E. coli* strain in India. In p52148_NDM-5, the *bla*_NDM–__5_ gene was found in a MDR region of 28400 bp in size (nts 105368-121872 and 1-11895 in GenBank accession no. CP050384). In both plasmids, p47733_NDM-5 and p52148_NDM-5, the genetic environment of *bla*_NDM–__5_ was identical (Figure 2). The *aadA2*, *dfrA12*, *sul1*, *mphA* and *tetA* resistance genes were also identified in the MDR region of p52148_NDM-5. The MDR region of p52418_NDM-5 was inserted downstream the *pemIK* operon, as it was also observed in p47733_NDM-5.

Finally, in all four carbapenemase-producing isolates, a diverse number of additional replicons were identified (Table 2). Some of these replicons were associated with important resistance determinants, like *bla*_CTX–M–__15_, *arr-2* and *ermB*.

## Discussion

Previous studies have reported the spread of *bla*_OXA–__48_-like and *bla*_NDM_-like genes in *Enterobacterales* recovered from Czech hospitals (Skalova et al., 2017; Paskova et al., 2018). However, based on our knowledge, this study reports the first description of OXA-244-producing *Enterobacterales* isolated from Czech hospitals. Both OXA-244-producing isolates, also expressed NDM-1 or NDM-5 MβLs. Additionally, two *K. pneumoniae* isolates, co-producing OXA-181 and NDM-type carbapenemases, were identified. Overall, horizontal gene transfer is the main mechanism involved in the dissemination of *bla*_NDM_-like and *bla*_OXA–__48_-like genes, but certain clones have been associated with the spread of these resistance genes (Pitout et al., 2019). Carbapenemase-producing *K. pneumoniae* and *E. coli* isolates, characterized during this study, belonged to clones (STs 11, 15, and 147 in *K. pneumoniae*, and ST167 in *E. coli*; Table 2), which have been previously characterized as “high risk clones,” and have been reported in association with these carbapenem-resistance mechanisms (Woodford et al., 2011; Pitout et al., 2019). In all cases, carbapenemase-producers were recovered from patients with travel history or previous hospitalization abroad. The endemicity of OXA-181 and NDM-5 carbapenemases among *Enterobacterales* isolated in the Indian subcontinent has been reported in several studies (Lascols et al., 2013; Krishnaraju et al., 2015; Ahmad et al., 2019; Pitout et al., 2019). Interestingly, a recent report from the United States described the characterization of a ST147 *K. pneumoniae* harboring *bla*_NDM–__5_ and *bla*_OXA–__181_ from a patient, who was previously hospitalized in India (Rojas et al., 2017). Also, North African countries, and especially Egypt, represent a geographical region, where *bla*_OXA–__48_-like and/or *bla*_NDM_-like genes are highly disseminated among *Enterobacterales* (Tafoukt et al., 2017; Soliman et al., 2020a,b). Studies from Italy have described the import of NDM-1-producing *K. pneumoniae* isolates from Egypt (Principe et al., 2016; Nucleo et al., 2020). Additionally, the import of OXA-244-producing *E. coli* isolates from countries in Northern Africa was observed in a surveillance from Denmark (Hammerum et al., 2020). Finally, two studies have documented the spread of NDM-1-producing *K. pneumoniae* isolates in Mauritius (Poirel et al., 2012; Holman et al., 2017), speculating a link with India, due to the geographical and cultural links between the two countries. In 2018, another study described the characterization of a *K. pneumoniae* isolate co-producing NDM-1 and OXA-181 carbapenemases, recovered from a patient, who had previously went to Mauritius (Allyn et al., 2018). These findings highly underline that import of carbapenemase-producing isolates via travel or/and hospitalization abroad could represent a risk for a further dissemination of these isolates in Czech hospitals. However, epidemiological data don’t confirm the scenario regarding the spread of *Enterobacterales* co-producing NDM- and OXA-48-like carbapenemases in Czech hospitals (Hrabak, unpublished results).

Although the main limitation of this study was the small number of *Enterobacterales* isolates co-producing OXA- and NDM-type carbapenemases, which were collected in clinical microbiology laboratories, analysis of WGS data revealed that all four isolates harbored a huge variety of genes conferring resistance to several categories of antibiotics. Additionally, inspection of contigs carrying carbapenemase-encoding genes showed that different genetic structures and replicon types were involved in the dissemination of these resistance determinants. These contigs also included a huge variety of insertion sequences that might be involved in the organization of MDR regions, conferring resistance to several antibiotic categories thus, limiting therapeutic options. Thus, in addition to different combinations of carbapenemase-encoding genes, the variability of replicon types, genetic structures, resistance genes and mobile elements observed among the studied isolates indicated the genetic plurality involved in the acquisition and dissemination of determinants encoding OXA/NDM carbapenemases.

## Data Availability Statement

The datasets presented in this study can be found in online repositories. The names of the repository/repositories and accession number(s) can be found below: https://www.ncbi.nlm.nih.gov/genbank/, CP050360–CP050384.

## Author Contributions

CP and IB played an important role in interpreting the results and in writing the manuscript. KC, LK, and JH helped to acquire data. KC, LK, VM, and IB carried out experimental work. CP supervised the experiments and revised the final manuscript, which was approved by all authors.

## Conflict of Interest

The authors declare that the research was conducted in the absence of any commercial or financial relationships that could be construed as a potential conflict of interest.

## Footnotes

1 http://www.eucast.org/

2 http://darlinglab.org/mauve/mauve.html

3 https://inkscape.org/
