# Supplementary material for: Genetic Plurality of OXA/NDM-Encoding Features Characterized From Enterobacterales Recovered From Czech Hospitals
Source: Front Microbiol. 2021 Feb 9;12:641415. doi: 10.3389/fmicb.2021.641415 (PMC7900173; doi:10.3389/fmicb.2021.641415)
Supplement: Supplementary file 1 [file Presentation_1.PPTX]

## Slide 1
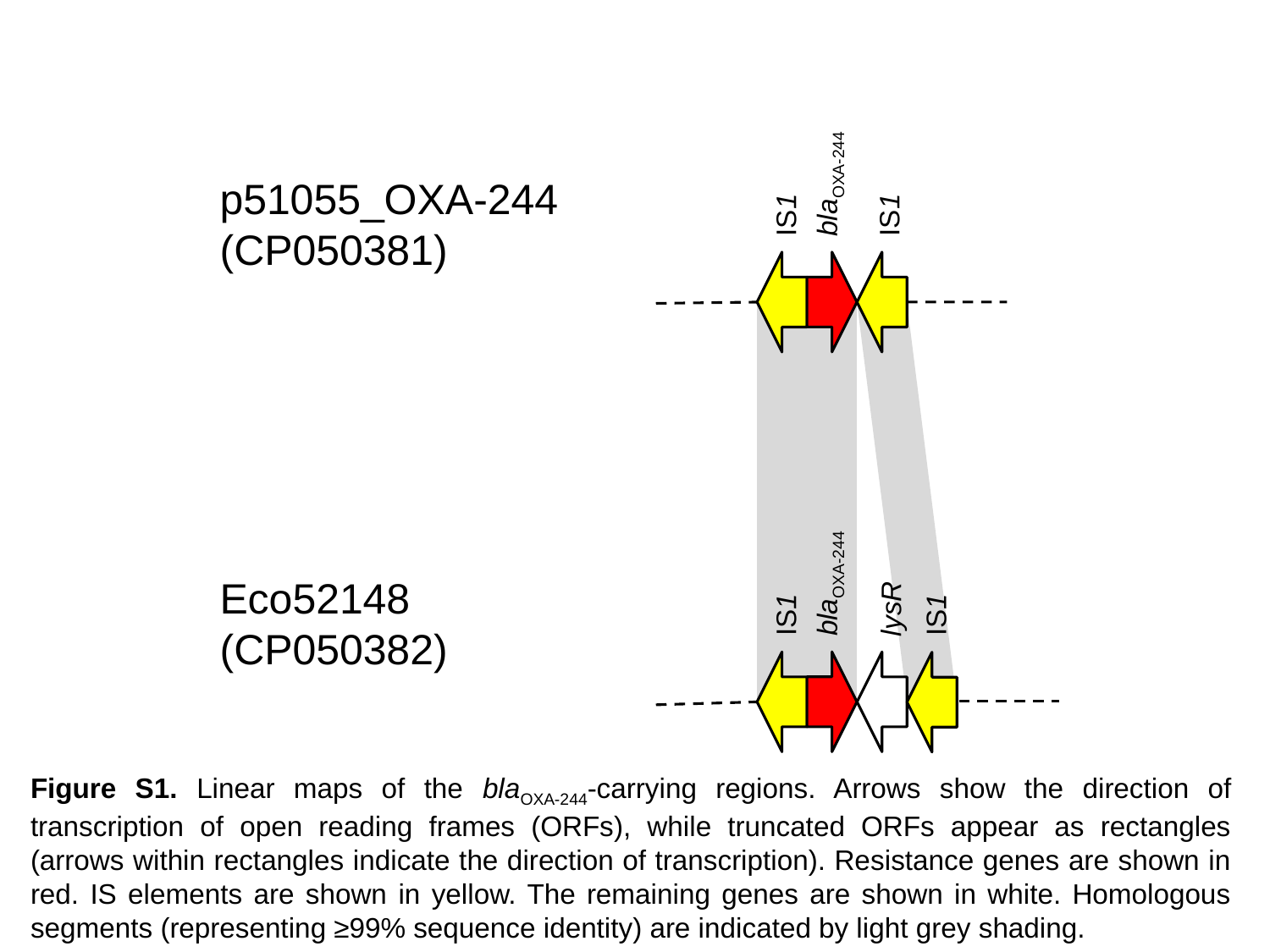

IS1
blaOXA-244
IS1
p51055_OXA-244 (CP050381)
IS1
blaOXA-244
IS1
lysR
Eco52148 (CP050382)
Figure S1. Linear maps of the blaOXA-244-carrying regions. Arrows show the direction of transcription of open reading frames (ORFs), while truncated ORFs appear as rectangles (arrows within rectangles indicate the direction of transcription). Resistance genes are shown in red. IS elements are shown in yellow. The remaining genes are shown in white. Homologous segments (representing ≥99% sequence identity) are indicated by light grey shading.
