# Supplementary material for: Genetic Plurality of OXA/NDM-Encoding Features Characterized From Enterobacterales Recovered From Czech Hospitals
Source: Front Microbiol. 2021 Feb 9;12:641415. doi: 10.3389/fmicb.2021.641415 (PMC7900173; doi:10.3389/fmicb.2021.641415)
Supplement: Supplementary file 2 [file Presentation_2.PPTX]

## Slide 1
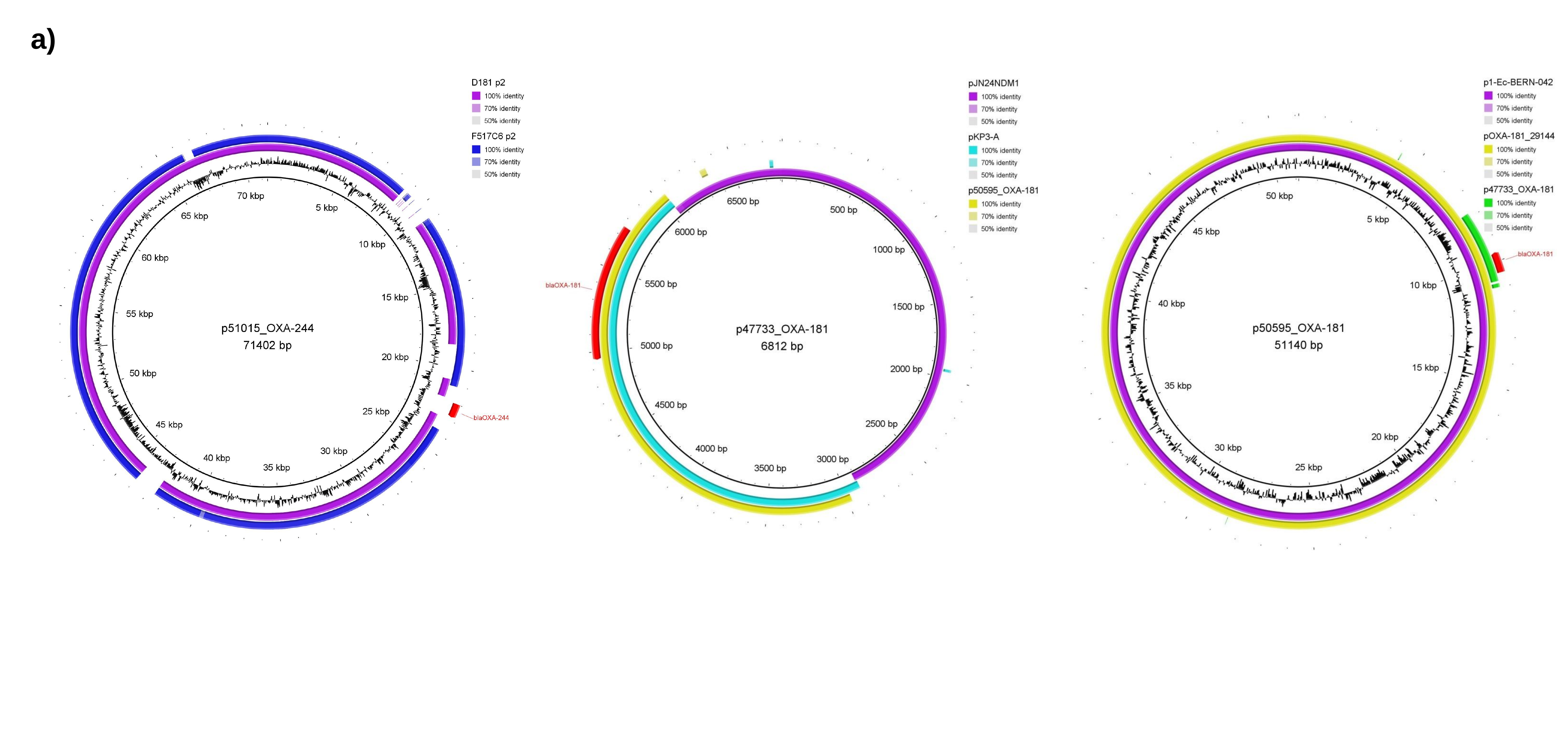

a)

## Slide 2
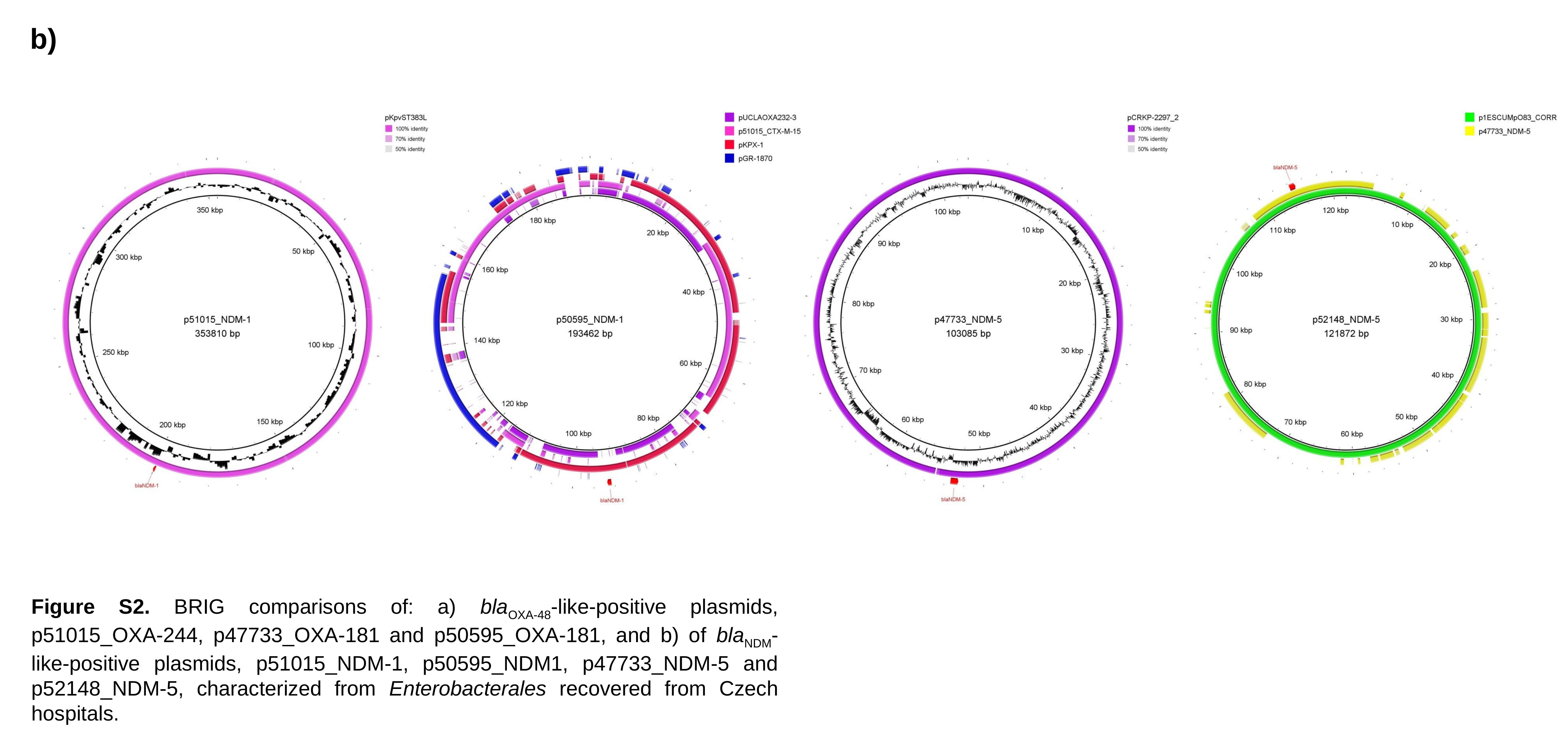

b)
Figure S2. BRIG comparisons of: a) blaOXA-48-like-positive plasmids, p51015_OXA-244, p47733_OXA-181 and p50595_OXA-181, and b) of blaNDM-like-positive plasmids, p51015_NDM-1, p50595_NDM1, p47733_NDM-5 and p52148_NDM-5, characterized from Enterobacterales recovered from Czech hospitals.
