# Supplementary material for: Genetic Plurality of OXA/NDM-Encoding Features Characterized From Enterobacterales Recovered From Czech Hospitals
Source: Front Microbiol. 2021 Feb 9;12:641415. doi: 10.3389/fmicb.2021.641415 (PMC7900173; doi:10.3389/fmicb.2021.641415)
Supplement: Supplementary file 3 [file Presentation_3.PPTX]

## Slide 1
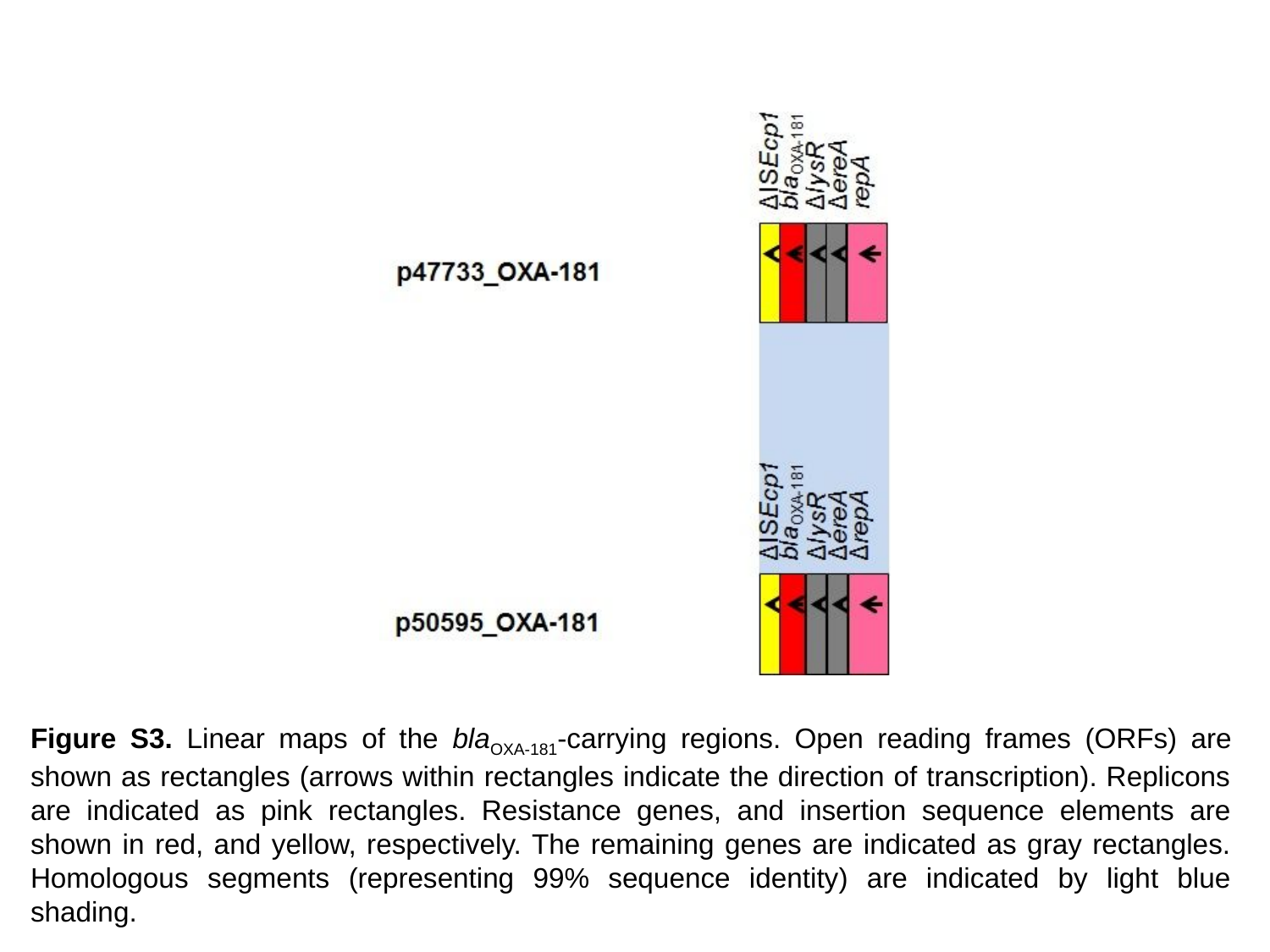

Figure S3. Linear maps of the blaOXA-181-carrying regions. Open reading frames (ORFs) are shown as rectangles (arrows within rectangles indicate the direction of transcription). Replicons are indicated as pink rectangles. Resistance genes, and insertion sequence elements are shown in red, and yellow, respectively. The remaining genes are indicated as gray rectangles. Homologous segments (representing 99% sequence identity) are indicated by light blue shading.
