# Supplementary material for: Genetic Plurality of OXA/NDM-Encoding Features Characterized From Enterobacterales Recovered From Czech Hospitals
Source: Front Microbiol. 2021 Feb 9;12:641415. doi: 10.3389/fmicb.2021.641415 (PMC7900173; doi:10.3389/fmicb.2021.641415)
Supplement: Supplementary file 4 [file Presentation_4.PPTX]

## Slide 1
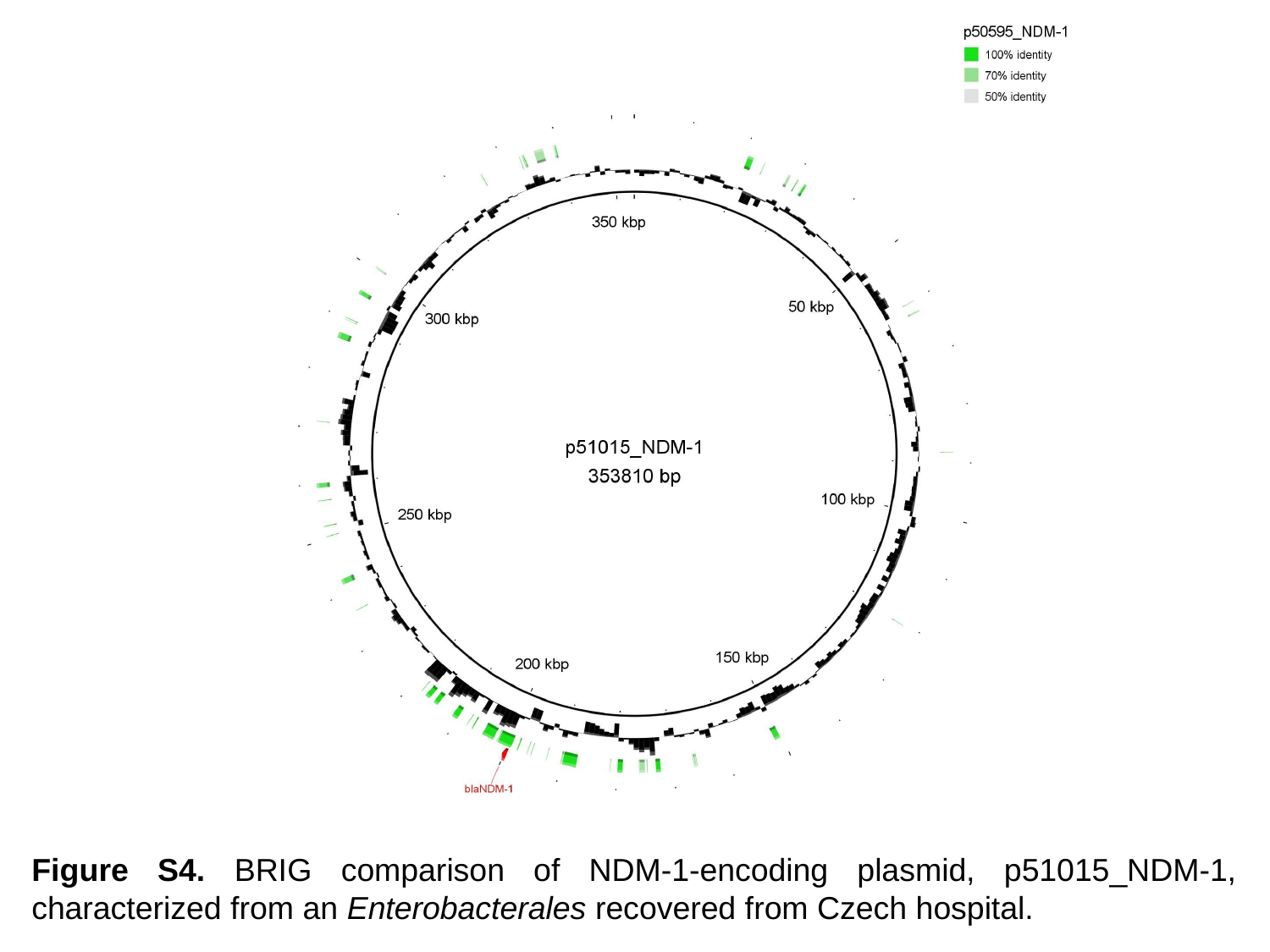

Figure S4. BRIG comparison of NDM-1-encoding plasmid, p51015_NDM-1, characterized from an Enterobacterales recovered from Czech hospital.
