# Supplementary material for: Genetic Plurality of OXA/NDM-Encoding Features Characterized From Enterobacterales Recovered From Czech Hospitals
Source: Front Microbiol. 2021 Feb 9;12:641415. doi: 10.3389/fmicb.2021.641415 (PMC7900173; doi:10.3389/fmicb.2021.641415)
Supplement: Supplementary file 5 [file Table_1.DOC]

**Table S1.** Susceptibility profiles of carbapenemase-producing transconjugants, obtained during the study.

| **Isolate** | **MIC(mg/L)** | | | | | | | | | | | | | |
| --- | --- | --- | --- | --- | --- | --- | --- | --- | --- | --- | --- | --- | --- | --- |
|  | **Amp** | **Pip** | **Tzp** | **Ctx** | **Caz** | **Mem** | **Etp** | **Gm** | **Amk** | **Tm** | **Net** | **Tet** | **Tgc** | **Cip** |
| *E. coli* A15  Kpn47733 | >128 | 128 | 128 | >8 | >16 | 2 | >2 | >32 | >64 | >8 | >16 | 2 | 0.125 | <0.06 |
| *E. coli* A15  Kpn50595 | >128 | >128 | >128 | >8 | >16 | 4 | >2 | >32 | >64 | >8 | >16 | 2 | 0.125 | >8 |
| *E. coli* A15  Kpn51015 | >128 | 128 | 64 | >8 | >16 | 4 | >2 | >32 | >64 | >8 | >16 | 2 | 0.125 | >8 |
| *E. coli* A15  Eco52148 | >128 | >128 | >128 | >8 | >16 | >16 | >2 | 0.5 | 0.5 | 0.5 | 0.5 | 32 | 0.125 | >8 |

MIC, minimum inhibitory concentration; Amp, ampicillin; Pip, piperacillin; Tzp, piperacillin-tazobactam; Ctx, cefotaxime; Caz, ceftazidime; Mem, meropenem; Etp, ertapenem; Gm, gentamicin; Amk, amikacin; Tm, tobramycin; Net, netilmicin; Tet, tetracycline; Tgc, tigecycline; Cip, ciprofloxacin.
